# Compliance with birth dose of Hepatitis B vaccine in high endemic and hard to reach areas in the Colombian amazon: results from a vaccination survey

**DOI:** 10.1186/s12913-016-1542-z

**Published:** 2016-07-21

**Authors:** Luz Angela Choconta-Piraquive, Fernando De la Hoz-Restrepo, Carlos Arturo Sarmiento-Limas

**Affiliations:** Public Health Department, Universidad Nacional de Colombia, Bogotá, Colombia

**Keywords:** Hepatitis B, Vaccine, Vaccination coverage, Birth dose, Colombia, Amazon, Rural

## Abstract

**Background:**

Hepatitis B vaccination was introduced into the Expanded Program of Immunization in Colombia in 1992, in response to WHO recommendations on hepatitis B immunization. Colombia is a low endemic country for Hepatitis B virus infection (HBV) but it has several high endemic areas like the Amazon basin where more than 70 % of adults had been infected. A cross- sectional study was carried out in three rural areas of the Colombian Amazon to evaluate compliance with the recommended schedule for hepatitis B vaccine in Colombian children (one monovalent dose given in the first 24 h after birth + 3 doses of a pentavalent containing Hepatitis B. (DPT + Hib + Hep B).

**Methods:**

A household survey was conducted in order to collect vaccination data from children aged from 6 months to <8 years. Vaccination status was related to sociodemographic data obtained from children caretakers.

**Results:**

Among 938 children above 6 months and < 8 years old studied, 79 % received a monovalent dose of hepatitis B vaccine, but only 30.7 % were vaccinated in the first 24 h after birth. This proportion did not increase by age or subsequent birth cohorts. Coverage with three doses of a DTP-Hib-HepB vaccine was 98 %, but most children did not receive them according to the recommended schedule. Being born in a health facility was the strongest predictor of receiving a timely birth dose.

**Conclusions:**

This study suggests that more focused strategies on improving compliance with hepatitis B birth dose should be implemented in rural areas of the Amazon, if elimination of perinatal transmission of HBV is to be achieved. Increasing the proportion of newborns delivered at health facilities should be one of the priorities to reach that goal.

**Electronic supplementary material:**

The online version of this article (doi:10.1186/s12913-016-1542-z) contains supplementary material, which is available to authorized users.

## Background

Perinatal transmission of hepatitis B virus ends in chronic hepatitis B infection in 80 to 90 % of the children exposed to the virus, while less than 1 % of newborns develop the acute form of the disease [1, 2]. Mathematical models estimate that 21 % of all hepatitis B virus (HBV) related deaths are attributable to infection in the perinatal period, and 48 % to infection in early childhood. Vaccinating 50–90 % of newborns would prevent 77 to 84 % of Hepatitis related deaths because it reduces by 70 to 95 % the risk of HBV transmission from mother to child [3]. In 1992, WHO recommended universal vaccination against HBV due to the high burden of the HBV-related diseases worldwide and later, in 2004, it endorsed the use of a birth dose to prevent perinatal transmission [1]. Although universal hepatitis B vaccination was introduced in Latin America countries (LAC) since mid-1990, there is a paucity of information on the quality of the vaccination process and its impact on HBV transmission across the region [4]. Colombia has several high endemic spots placed in deep rural areas including the Amazon basin. In the pre-vaccination era, carriage of HBV surface antigen (HBsAg) was above 8 % and outbreaks of fulminant Hepatitis were common in those places [5]. In Colombia, universal vaccination against HBV for children was introduced in 1992 in a 0-1-2 schedule with a monovalent hepatitis B vaccine. In 1999, a field evaluation of the vaccination program found a reduction in HBV infection, but vaccination coverage with birth dose was low (25 %) and subsequent doses lagged compared to DPT vaccine [6]. To improve prevention, in 2001 Colombia modified the HBV vaccine schedule, switching from 3 monovalent doses to 3 doses of a pentavalent vaccine (diphtheria, tetanus, pertussis, *Haemophilus influenzae* type b and hepatitis B) plus one monovalent birth dose.

In 2011, a serological survey was carried out in a sample of children living in rural areas of the Colombian Amazon aiming to assess the prevalence of HBV infection and HBsAg carriage after the change of HBV vaccine schedule. This paper describes the findings related to compliance with the vaccination schedule and factors related to timely vaccination.

## Methods

### Study design

A population-based household survey was conducted to assess vaccination coverage and its characteristics in the Colombian Amazon department among children above 6 months and < 8 years old. Age was restricted because only children < 8 years. old were exposed to the vaccination schedule under study.

### Study population

The municipalities included in the survey were Leticia, Puerto Nariño, and Tarapacá. Leticia is the capital of the department of the Amazon with an estimated population of 39,667 inhabitants with 37 % living in rural areas. Puerto Nariño is the second most populated municipality with 7574 inhabitants, 73.3 % living in the rural area. Tarapacá is a rural area with small human settlements scattered along the banks of the Putumayo and the Cothue rivers with a population of 3992 inhabitants. Leticia and Puerto Nariño were chosen because they have the largest population in the department. Tarapacá was selected as representative of the most isolated rural areas where a substantial proportion of the Amazon population lived.

### Sample size

The main goal of this study was to assess the prevalence of HBV infection and HBsAg carriage among children living in rural areas of the Amazon. Sample size was calculated to assess an HBsAg carriage prevalence of 1 % within a confidence limit of 0.5 and 95 % of confidence level. The number needed to fulfill those assumptions was estimated to be ~1300 children. At the end, more than 900 children < 8 years were available for the analysis on vaccine coverage. That sample size would be enough to estimate a vaccination coverage of 80 % with confidence limits of 8 %.

### Sampling criteria

For Leticia and Puerto Nariño, only rural areas settled along the Amazon river (pop < 8 years ≈ 2700 according to EPI field census) were included in the study because HBsAg carriage was considerably higher and vaccination was lower than in urban settlements or rural areas placed elsewhere. [6–8]. Most people belong to aboriginal tribes and live under the poverty line with low access to health and sanitation services. In Leticia 12 of 21 villages were included; in Puerto Nariño 17 villages out of 20 were included and in Tarapacá, 8 communities out of 13 were included. Six communities were excluded because of a very low population (only one family) or language constraints. One community (YAGUAS) rejected to participate in the study.

Community and tribal leaders in every municipality were invited to a meeting, conducted by the researchers, where they were briefed about the following issues: objectives of the study, importance of hepatitis B infection for their communities, relevance of the vaccination program for hepatitis B control, and the relevance of monitoring infection prevalence periodically.

Once a community approved to participate in the study, field workers started household visits in order to find out the information on vaccination status of every child who match the inclusion criteria within the household. All children born from 2004 onwards, present at the time of the visit and whose parents consented to participate in the study were included. A household was defined as all those sleeping under the same roof regularly. The field work team was composed of bacteriologists, nurses, and a field coordinator. The survey was conducted between July 2011 and March 2012.

For Leticia and Puerto Nariño participants were selected from villages settled along the banks of the Amazon River and the Loretoyaco River. It was restricted to those settlements because they tend to have lower coverages and higher HBV prevalence than areas accessible by road [6].

### Selection of participants

In the villages selected for the study every household was visited and parents invited to participate in the study. All eligible children found in any given household were included in the study if their caretakers approved to participate. Households were visited starting from the farthest to the nearest to the center of the community.

### Hepatitis B vaccination data

Vaccination dates were recorded from vaccination cards. When vaccination cards were not available or difficult to read the Expanded Program on Immunization (EPI) databases were consulted. These databases are updated by the EPI nurses and contain vaccination data from every child living in rural areas. Recall data was not used. A timely birth dose should be given in the first 24 h after delivery as recommended by the WHO [1]; however in our study, vaccination cards show dates of birth and administration of monovalent HBV dose but lack data on both, exact hour of birth or hour of vaccine administration. Therefore, a timely birth dose was defined as a monovalent dose of the hepatitis B vaccine administered on the same date of delivery or the day after. A timely vaccinated child was described as having a timely monovalent dose plus 3 doses of pentavalent vaccine given at 2, 4 and 6 months with at least 30 days between each dose.

The research protocol was approved by the medical ethics committee of the school of medicine, Universidad Nacional de Colombia, October 8, 2010. All participants were informed of the purpose and procedures of the study and all the parents or legal guardians gave their signed consent for their children to participate.

### Independent variables

The following variables were collected : 1) vaccination status, 2) mother’s education level, 3) level of wealth measured by ownership of boat or electric appliances, 4) overcrowding (defined as more than 5 people per room), 5) mother’s ethnic group, 5) children sex, 7) site of birth (home, health care facility other), 8) person attending birth, 9) child’s health insurance regime for which there were three options: belonging to the Contributive regime or to the Subsidized regime. In Contributive regime workers in the formal sector pay a monthly sum and have access to a wider health care set of services. The Subsidized regime is designed for informal workers who pay a lower sum and are entitled to a more restrictive health care portfolio. It should be emphasized that hepatitis B vaccine is provided freely by the Colombian M o H and every child has the right to be vaccinated regardless his/her insurance scheme.

### Statistical analysis

Information was entered into databases using EpiInfo 3.5.3 ®, and statistical analysis were performed in SPSS 19® and Stata 11.1 ® software. Coverage with the birth dose, coverage with a timely birth dose and mean time to the monovalent hepatitis B vaccine dose were described by birth cohort. An inverse Kaplan-Meier curve was used to describe coverage and timeliness of vaccination with the monovalent birth dose. To identify predictors of receiving a timely birth dose of monovalent hepatitis B vaccine, bivariate analysis were conducted relating it to the following independent variables: municipality, mother’s education level, household wealth, crowding, ethnic group, child sex, site of birth, and child’s health insurance. Odds Ratios (OR), 95 % confidence intervals, and *p* values were estimated to assess the strength and statistical significance of those associations. All independent variables found related to timely monovalent dose (*p* < 0.1) were included in a multivariate model (unconditional logistic regression) using a stepwise approach.

Children were selected using villages as the sampling unit, so children in a specific village tended to be similar, but different, in a range of characteristics, compared to children in a different village. This “clustering effect” may lead to underestimate the magnitude of the variance of the OR estimates. Hence, a mixed-effects logistic regression analysis was performed to control for that cluster effect. The model was built using the same strategies described for the unconditional logistic regression. Results from both models were compared and, if no important difference was observed, only results from the simple model was presented. Timely vaccination with pentavalent was assessed in a similar way.

To assess the direction and magnitude of potential selection biases introduced by children without vaccination card, additional analysis were conducted. Children without a valid vaccination card, whose vaccination record could not be recovered from EPI local registries, were classified as no vaccinated and were included in the analysis. Then, changes in ORs values were noted and appraised if they occur. To assess the influence of potential information bias, an additional analysis was conducted classifying as unvaccinated the 30 children without vaccination card.

## Results

A total of 938 children aged 6 months to 8 years were included and vaccination card was available for 97 % of them. Children in the study represented between 24 and 48 % of the total number of eligible children living in the rural areas of the surveyed municipalities. The larger proportion of rural children, compared to the total rural population in each municipality, was selected from Puerto Nariño and Tarapacá, 48 and 45 % respectively. In Leticia, 24 % out of all children living in rural areas were surveyed, Less than 1 % of parents invited to participate refused. One community, representing less than 10 % of the total population living in the rural settlements along the Amazon River, declined to participate in the study.

**Fig. 1:**
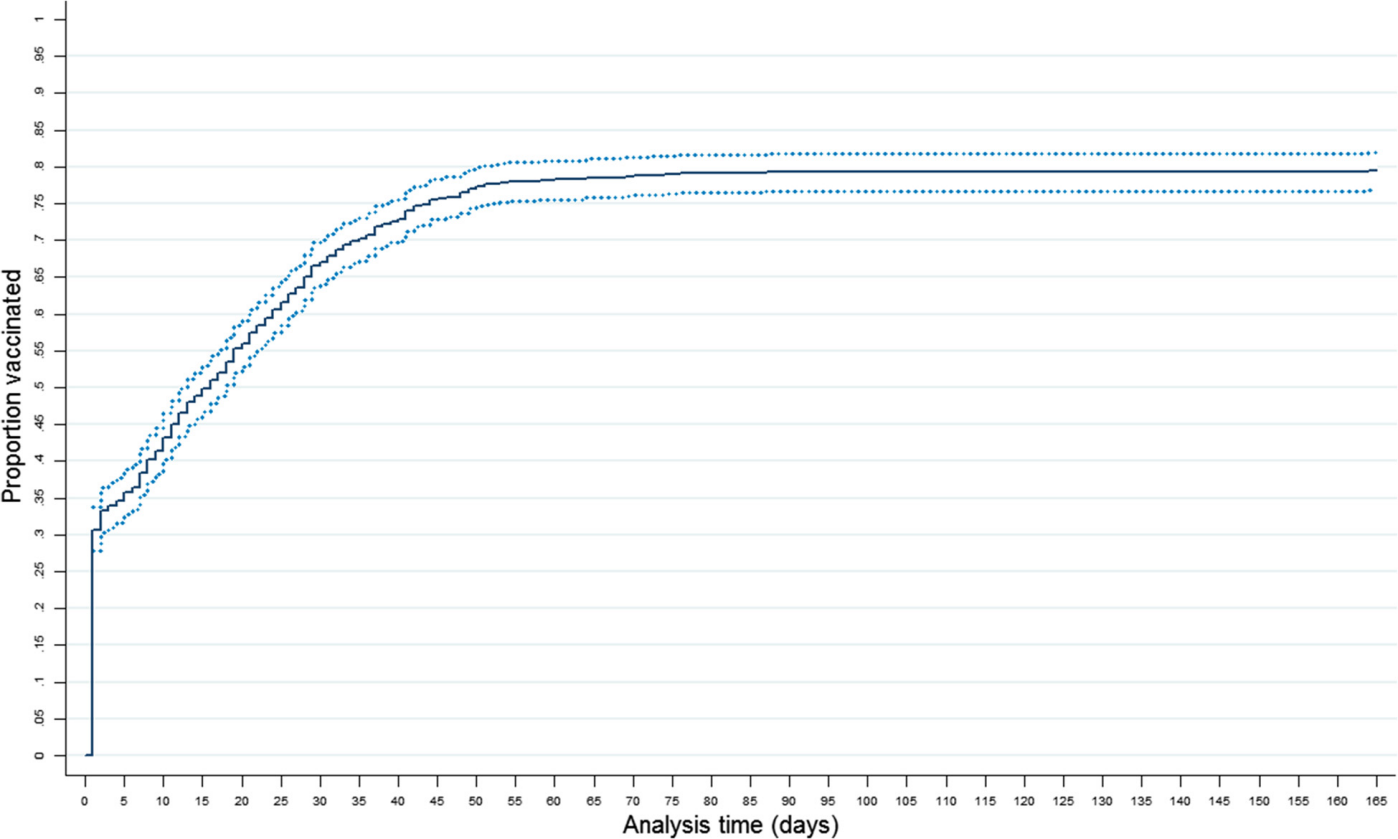
Proportion of children vaccinated with the hepatitis B birth dose. Leticia, Nariño, Tarapacá; Amazon department, Colombia.1- SKM (t) representing the proportion of children being vaccinated with a monovalent hepatitis B dose at any given age (t). The last children vaccinated received a monovalent hepatitis B dose 164 days after being born

**Fig. 2:**
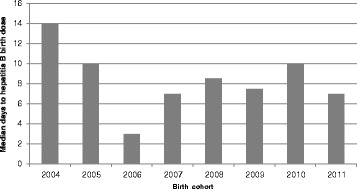
Median days to hepatitis B birth dose according to the birth cohort. Leticia, Puerto Nariño, Tarapacá; Amazon department, Colombia

**Fig. 3:**
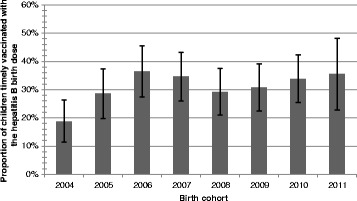
Proportion of children receiving a timely hepatitis B birth dose by birth cohort. Leticia, Puerto Nariño, Tarapacá; Amazon department, Colombia. Error bars represent 95 % confidence intervals

The mean age of the participants was 3.7 years (CI 95 % 3.5–3.8), and the median was 4 years, IQR (interquartile range) of 4. Males represented 51.6 % of the sample. Most children, 79.4 % (747) (CI 95 % 77–82.3 %), received a hepatitis B monovalent dose, only 30.7 % (*n* = 288) filled the definition for a timely birth dose. On average, children received the monovalent dose 14 days after delivery (95 % CI 12.8–15.3) with a median of 8 days, IQR = 23. Figure 1 shows the inverse survival curve of the progression of the vaccination. The median time from delivery to birth dose is presented in Figs. 2 and 3 depicts the proportion of children with a timely birth dose by birth cohort. The highest coverage with a timely birth dose was observed for children born in 2006 (46 %), Fig. 2 shows that the median time to receive the birth dose in 2006 was 3 days.

Table 1 displays the results of the bivariate and multivariate analysis relating the characteristics of vaccinated children with timeliness of hepatitis B monovalent dose. Bivariate analysis showed that place of birth (hospital), municipality, race, wealth, and overcrowding were associated with receiving a timely birth dose of hepatitis B vaccine.

**Table 1:** Selected variables associated with timely vaccination with the hepatitis B birth dose. Leticia, Puerto Nariño, Tarapacá; Amazon department, Colombia 2011–2012

			Birth dose^a^			
	*n*	Missing	Vaccinated	Not vaccinated	Crude OR	Adjusted OR^b^
					*p* value	*p* value
Sex			*p* = 0.207			
Female	453	2	28.7 %(130)	71.3 %(323)		
Male	483		32.5 %(157)	67.5 %(326)		
	936					
Municipality			*p* < 0.0001		*p* < 0.0001	*p* < 0.001
Leticia	334	0	38.6 %(129)	61.4 %(205)	–	–
Puerto Nariño	397		25.9 %(103)	74.1 %(294)	0.6 (0.4–0.8)	0.6 (0.4–0.8)
Tarapacá rural	156		16 % (25)	74.1 % (131)	0.3 (0.2–0.5)	0.3 (0.2–0.6)
Tarapacá main village	51		60.8 %(31)	39.2 %(20)	2.5 (1.3–4.5)	1.0 (0.5–2.2)
	938					
Place of birth			*p* < 0.0001		*p* < 0.0001	*p* < 0.001
Health care facility	320	9	72.5 %(232)	27.5 %(88)	28.2 (19.4–41.1)	27.2 (18.5–40)
Other	609		8.5 %(52)	91.5 %(557)	–	
	929					
Ethnic group of the mother			*p* = 0.008		*p* = 0.009	
Ticuna	714	0	28.4 %(203)	71.6 %(511)	0.4 (0.2–0.8)	
Other	180		35.6 %(64)	64.4 %(116)	0.6 (0.3–1.2)	
None	44		47.7 %(21)	52.3 %(23)	–	
	938					
Appliances in the household (Boat, refrigerator, television or radio)			*p* < 0.0001		*p* < 0.0001	
One or more	558	5	36.7 %(205)	63.3 (353)	2.1 (1.6–2.8)	
None	375		21.6 %(81)	78.4 %(294)	–	
	933					
Birth assisted by			*p* < 0.0001		*p* < 0.0001	
doctor or nurse	318	9	72.6 %(231)	27.4 %(87)	28 (19.2–40.6)	
Other	611		8.7 %(53)	91.3 %(558)	–	
	929					
Overcrowding			*p* = 0.001		*p* = 0.001	
0–5 people/room	645	12	33.6 %(217)	66.4 %(428)	1.7 (1.2–2.3)	
>5 people/room	281		23.1 %(65)	76.9 %(216)	–	
Health insurance			*p* = 0.01		*p* = 0.013	
None	46	17	80.4 %(37)	19.6 %(9)	0.2 (0.07–0.6)	
Subsidized	849		30.5 %(259)	69.5 %(590)	0.4 (0.2–0.8)	
Contributive (paid by the affiliated)	26		53.8 %(14)	46.2 %(12)	–	
	921					
Mother’s education (mean years)			*p* < 0.0001		*p* < 0.0001	
	938	0	4.23	3.33	1.1 (1.0–1.1)	

^a^Timely birth dose defined as a monovalent dose of the hepatitis B vaccine administered on the same date of delivery or the day after

^b^logistic regression

In the final multivariable model, only place of birth and municipality remained associated with receiving a timely birth dose of hepatitis B. (Table 1). The variable “birth assisted by” was dropped from the model because it was highly correlated with the place of birth. Children born at health facilities had a disproportionate chance of receiving a timely birth dose compared to those born elsewhere. (OR 27.2, 95 % CI 18.5–40). Living in the main village of Tarapacá (where the hospital is located) or in the rural areas of Leticia increased the chance of getting a timely birth dose of hepatitis B monovalent vaccine.

The model where the children without a vaccination card were classified as unvaccinated yielded similar results. The place of birth was still the strongest associated variable (OR22.6 CI 95 % 15–34.1) with not being vaccinated. A mixed effects model was run using the same variables than the conventional logistic regression. No differences were found for the results of both models and goodness of fit was better for the conventional logistic regression model (Likelihood Ratio test = 8.3, *p* = 0.002).

### Vaccination with the pentavalent vaccine

Coverage was 99.7 % (95 % CI 99.1–99.9 %) for the first dose, 99.3 % (95 % CI 98.5–99.7 %) for the second and 98.2 % (95 % CI 97.1–98.9 %) for the third. Most children, 78.3 %, received a monovalent dose plus three pentavalent doses. The first pentavalent dose was received, on average, at 92.7 days (95 % CI 87.9–97.6), median of 74 and IQR (interquartile range) of 37; the second dose at 169 days (95 % CI 163–176), median of 144 and IQR of 57; the third dose was given at 251 days (95 % CI 242–260), median of 209 and IQR of 71. Only 15.7 % of the children were vaccinated as recommended by the WHO and the Colombian EPI guidelines. Timely vaccination with pentavalent increased among those living in a household with less than 5 people/room (OR 1.7 CI 95 % 1.2–2.5); born in a health facility (OR 1.5 CI 95 % 1.1–2.1); or having a more educated mother. (OR 1.085 CI 95 % 1.028–1.1/year of education).

## Discussion

This study shows that coverage with a timely birth dose of HBV vaccine is still low among high risk populations, which makes elimination of perinatal transmission very challenging for these areas. In 1999, a probabilistic sample of rural and urban children living in the Colombian Amazon, found that a timely birth dose only reached to 25 % of children born after the beginning of HBV vaccination. Though the methodology of the 1999 survey is different and caution should be exerted when comparing results, the 2011 study also found a low coverage with hepatitis B birth dose, suggesting a lasting low effectiveness of the primary health care system in the Amazon. [6]. Only two studies describing coverage with HBV birth dose have been published from Latin American countries.

Among children born in health facilities (*n* = 320), 94 % received the monovalent dose and 72.5 % got it timely. From the remaining children, 69 were vaccinated with the monovalent within 87 days after birth, and 19 did not receive it.. Nationwide, the Colombian EPI reports that at least 80 % of newborns received a monovalent hepatitis B dose, but there is no data on how many receive it within the first 24 h after birth. Our survey showed that although monovalent dose is reported on vaccination card as a birth dose, yet 50 % of children received it more than 8 days after birth. It suggests that the actual coverage with a birth dose of hepatitis B in Colombia may be lower than the official report.

The introduction of a pentavalent vaccine seems to have increased the proportion of children receiving three doses of hepatitis B. Before the introduction of pentavalent, the Colombian EPI reported that less than 80 % of children have received three doses of hepatitis B by the age of one year. Nowadays, more than 90 % of children across the nation received three doses of hepatitis B in the first year of age, and this increase is partially an impact of switching from monovalent to pentavalent. However, our study shows that most children received three doses of pentavalent but that they are delayed.

Our study have some methodological strengths. Information bias was avoided by using only written information on vaccination status either from vaccination cards or local EPI records. To avoid mistakes while filling study forms with vaccination data from EPI cards, field workers were intensively trained to check for inconsistencies between among vaccination dates and birth dates. Also, the database was programmed to check for similar inconsistencies. Less than 5 % of the records were found with such inconsistencies and most of them were corrected. Actions were taken to reduce potential selection bias. Meetings with leaders of the communities previous to the beginning of field work helped to improve participation rates. During the field work, households were visited in a pre-established order to avoid volunteering. As a result of those strategies, less than 1 % of the parents invited refused to take part in the study. Only one community, the Yaguas, rejected to participate in the study but they represent less than 10 % of the total population living in the rural settlements along the Amazon River, Therefore its impact on vaccine coverage estimation should be negligible.

There were also potential threats for validity. One of the most important was the likelihood of selection bias because villages were selected using a non-probabilistic sampling method. As described in methods, we were interested in studying those areas known to have a high prevalence of Hepatitis B infection where good vaccination practices are more critical. In spite of using a non-probabilistic sample, results are consistent with national estimates, as for the proportion of children receiving three doses of pentavalent. They are also consistent with international findings, as for the relation between being born in a health facility and the likelihood of getting a birth dose [9, 10].

Low birth dose coverage has been reported in other endemic countries such as China, India, and Cambodia, where it ranged from 3 to 36 % in rural areas [9, 10]. Only one study was found from other Latin American country, Brazil, where birth dose coverage was 40.4 % in 27 urban areas with an even lower coverage endemic regions [11]. Studies from China and Cambodia found similar factors associated with timely birth dose, being born at a health facility, parent’s awareness about hepatitis B vaccine birth dose, and mother’s education [9, 10]. Most children in Colombia (95.4 %) are delivered at health facilities and only 4.3 % are delivered at home. However, this proportion is higher for rural areas (12.1 %) and the Amazon has the highest proportion of non-institutional birth in the country (31 %) [12]. We found a higher proportion of home deliveries, 66 %, which could be attributed to our sample being drawn from rural areas.

In the present study, up to a third of children delivered at hospital did not receive a timely birth dose of hepatitis B. Other studies (Papua New Guinea, The Philippines, and Laos) found that factors associated to not receiving a timely birth dose within health facilities included: poor knowledge on vaccination policy, wrong contraindications, and lack of knowledge on levels of hepatitis B infection in the target population [13–15]. Out of pocket expenses seem to be an important barrier in developed countries [16].

Municipality predicted timely vaccination with hepatitis B birth dose. Children living in the main village of Tarapaca, where the local hospital is placed, and children from the rural areas of Leticia, the capital of the department, had a higher chance of being timely vaccinated. Differences by geographical area occur because vaccinating rural villages along the Amazonian rivers is quite expensive. Vaccines and supplies are shipped from Leticia by boat or plane, which is costly and unreliable, creating shortage of both elements. Geographical barriers have been identified in other developing countries too [17, 18].

There have been attempts to increase birth dose coverage using strategies that promote birth delivery at health facilities or improving the opportunity to receive a birth dose among those born at home. Using out- of -the -cold chain stored vaccines increased birth dose coverage for children delivered at home, without decreasing antibody response or increasing vaccine wastage. Initiatives to increase the proportion of births at hospital facilities have also succeeded to increase the proportion of children receiving birth dose properly [16, 19–22].

## Conclusions

In spite the low coverage with the birth dose and difficulties to comply with the recommended schedule, studies from the Amazon in Colombia showed that there have been dramatic reductions in the prevalence of infection and carriage after universal hepatitis B vaccination was introduced [8, 23, 24]. However, if elimination of perinatal transmission is to be reached in the Amazon it would be mandatory to increase the proportion of newborns receiving a timely birth dose. Current strategies should include the frequent training and retraining of health workers on catch-up schedules and on ways to increase the proportion of women giving birth at health care facilities.

## Abbreviations

CI, confidence interval; EPI, Expanded Program on Immunization; HBsAg, HBV surface antigen; HBV, Hepatitis B infection; IQR, interquartile range; LAC, Latin America countries; OR, Odds Ratios; WHO, World Health Organization

## Additional file

Additional file 1: Study database containing Hepatitis B vaccination data from participants, Leticia, Puerto Nariño, Tarapacá; Amazon department, Colombia 2011–2012. (XLSX 256 kb)
